# A multicenter, randomized, parallel-controlled clinical trial protocol to evaluate the safety and efficacy of irreversible electroporation compared with radiofrequency ablation for the treatment of small hepatocellular carcinoma

**DOI:** 10.1186/s12957-024-03614-z

**Published:** 2024-12-20

**Authors:** Chao Cheng, Min Xu, Jinhua Pan, Qiang Chen, Kai Li, Dong Xu, Xiang Jing, Qiang Lu, Hong Yang, Qiyu Zhao, Zhuang Deng, Tian’an Jiang

**Affiliations:** 1 Hepatobiliary and Pancreatic Intervention Center, The First Affiliated Hospital, Zhejiang University School of Medicine, Hangzhou, Zhejiang China; 2 Department of Ultrasound Medicine, The First Affiliated Hospital, Zhejiang University School of Medicine, Hangzhou, Zhejiang China; 3 Zhejiang CuraWay Medical Technology Co.,Ltd, Hangzhou, Zhejiang China; 4 Department of Ultrasound, The Third Affiliated Hospital of Sun Yat-sen University, Guangzhou, Guangdong China; 5 Zhejiang Cancer Hospital, Cancer Hospital of the University of Chinese Academy of Sciences, Hangzhou, Zhejiang China; 6 Institute of Basic Medicine and Cancer (IBMC), Chinese Academy of Sciences, Hangzhou, Zhejiang China; 7 Department of Ultrasound, Tianjin Third Central Hospital, Tianjin, 300170 China; 8 Department of Ultrasound, West China Hospital, Chengdu, Sichuan China; 9 Department of Medical Ultrasonics, First Affiliated Hospital of Guangxi Medical University, Nanning, Guangxi Zhuang Autonomous Region, 530021 P.R. China

**Keywords:** Irreversible electroporation (IRE), Radiofrequency ablation (RFA), HCC, Ablation, Protocol

## Abstract

**Background:**

At present, the main clinical application of local ablation therapy, such as radiofrequency ablation (RFA), is to heat the tissue to a certain temperature. However, high temperature will cause thermal damage. Irreversible electroporation (IRE) is a novel minimally invasive local ablation technology for tumors. By high-frequency pulse, the tumor cell membrane can be irretrievably perforated, resulting in the destruction of the intracellular environment, which can preserve important structures in the treatment area. However, there are no randomized controlled clinical trials comparing the efficacy of IRE with traditional local ablation in the treatment of liver cancer.

**Aims:**

This study aims to conduct a randomized controlled clinical trial comparing the efficacy of IRE with RFA in the treatment of liver cancer.

**Methods:**

We will conduct a multicenter, randomized, parallel-controlled non-inferiority clinical trial to compare the efficacy and safety of IRE and RFA for hepatocellular carcinoma (HCC). One hundred and ninety patients with HCC from five academic medical centers will be enrolled. The patients will be randomized into treatment arm (IRE) and control arm (RFA). The primary outcome is the progress -free survival (PFS) and the key secondary outcome is the Overall survival (OS).

**Results:**

Forty-eight patients had been recruited from 5 centers, of which, 33 patients (median age, 59.1 years) with 38 tumors had completed the 1-month follow-up and 21 patients have complete the 3-month follow up, with 2.3 months median follow up period. The mean largest tumor diameter is 3.9 cm. No end point was observed for PFS or OS in both groups, and the complete ablation rate was 100% in both groups. The lesions in the IRE group showed obvious shrinkage 1 month after procedure. One major adverse event (AE) was occurred in the control group.

**Conclusion:**

This is the first randomized controlled clinical trial to compare the clinical effects of IRE and RFA. The preliminary results suggest that both RFA and IRE are effective in the treatment of HCC, which can provide strong evidence for the use of IRE in HCC and provide more options for the treatment of patients with HCC.

**Clinical Trial Registration:**

ClinicalTrials. gov, identifier NCT05451160.

**Supplementary Information:**

The online version contains supplementary material available at 10.1186/s12957-024-03614-z.

## Introduction

Hepatocellular carcinoma (HCC) is the leading cancer in the world, which is ranked top six of the most common cancer and top three of the most common cancer death, with about 500,000 deaths worldwide each year [1]. Its incidence is rising faster than any other cancer [2, 3]. In recent years, percutaneous local ablation has been recognized as a potential treatment for small hepatocellular carcinoma (typically < 3–4 cm), supplemented by surgical resection and liver transplantation [4]. The commonly used ablation methods for HCC are thermal ablation, including radiofrequency ablation (RFA), microwave ablation (MWA), and cryoablation [5]. The goal of thermal ablation is to induce tumor coagulative necrosis at temperatures above 60 °C or below − 40°C [6]. These techniques can ablate tumor tissue and surrounding tissue [7]. However, many tumors cannot be treated with thermal ablation due to their hazardous location. Thermal ablation of tumors near large vessels is associated with a higher incidence of incomplete eradication (heat sink effect) [8–11]. In addition, these modalities can cause thermal damage to vital structures near the ablation area, which can easily lead to serious adverse events [12].

**Fig. 1:**
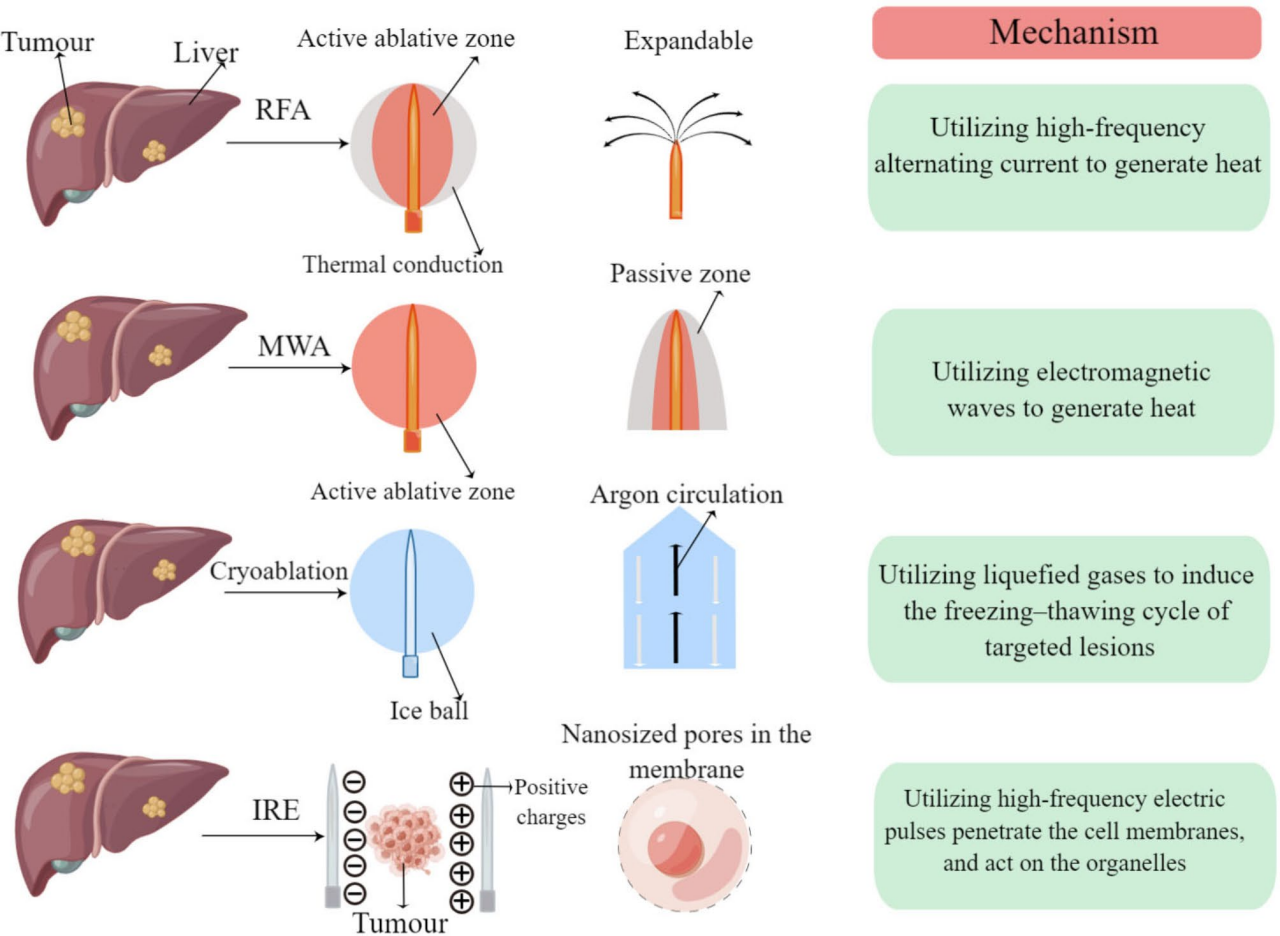
Mechanisms of local ablative methods for treatment of hepatocellular carcinoma RFA: radiofrequency ablation; MWA: microwave ablation; IRE: irreversible electroporation. This figure was drawn from Figdraw website (https://www.figdraw.com/static/index.html)

Irreversible electroporation (IRE) is a novel minimally invasive ablation technology for tumors. By high-frequency pulse, the tumor cell membrane can be irretrievably perforated at the nanometer level, resulting in the destruction of the intracellular environment, apoptosis and necrosis. Compared with traditional ablation techniques, it has unique advantages: good tissue selectivity, which can preserve important structures in the treatment area, clear boundaries between the ablation area and non-ablation area, short ablation time, necrosis caused by lack of heat and not affected by thermal/cold desorption of large blood vessels. Its safety and effectiveness have been widely verified in animal experiments [13–15]. Compared with thermal ablation, IRE does not affect the extracellular matrix, thereby maintaining the integrity of blood vessels and bile ducts. In addition, its effect is not affected by temperature changes and is not affected by the “heat sink effect” [16–20]. These characteristics make IRE attractive for the treatment of HCC, especially those at high-risk locations (the mechanisms of RFA, microwave ablation, cryoablation, and IRE are shown in Fig. 1).

IRE has been gradually applied in clinical practice worldwide and is expected to be an effective adjuvant therapy for patients with advanced liver cancer. Previous studies have compared the effects of IRE and RFA, but were all retrospective in nature and nonrandomized prospective study. For example, Thamtorawat et al. [21] retrospectively evaluated 42 patients with HCC who underwent IRE and RFA from January 2014 to September 2020 and compared the efficacy and safety of these treatments. Biliary tract adverse events were found in one (7.7%) patient in the IRE group and five (16.1%) patients in the RFA group. Although the study showed that the safety and efficacy of IRE were greater than those of RFA, it was retrospective in nature, preventing control of the consistency of baseline data between the two groups. Clinical trials comparing radiofrequency and IRE have not yet been reported. Therefore, we conduct a randomized controlled clinical trial to evaluate the efficacy and safety of IRE compared with RFA for HCC ablation.

## Materials and methods

### Study design

**Fig. 2:**
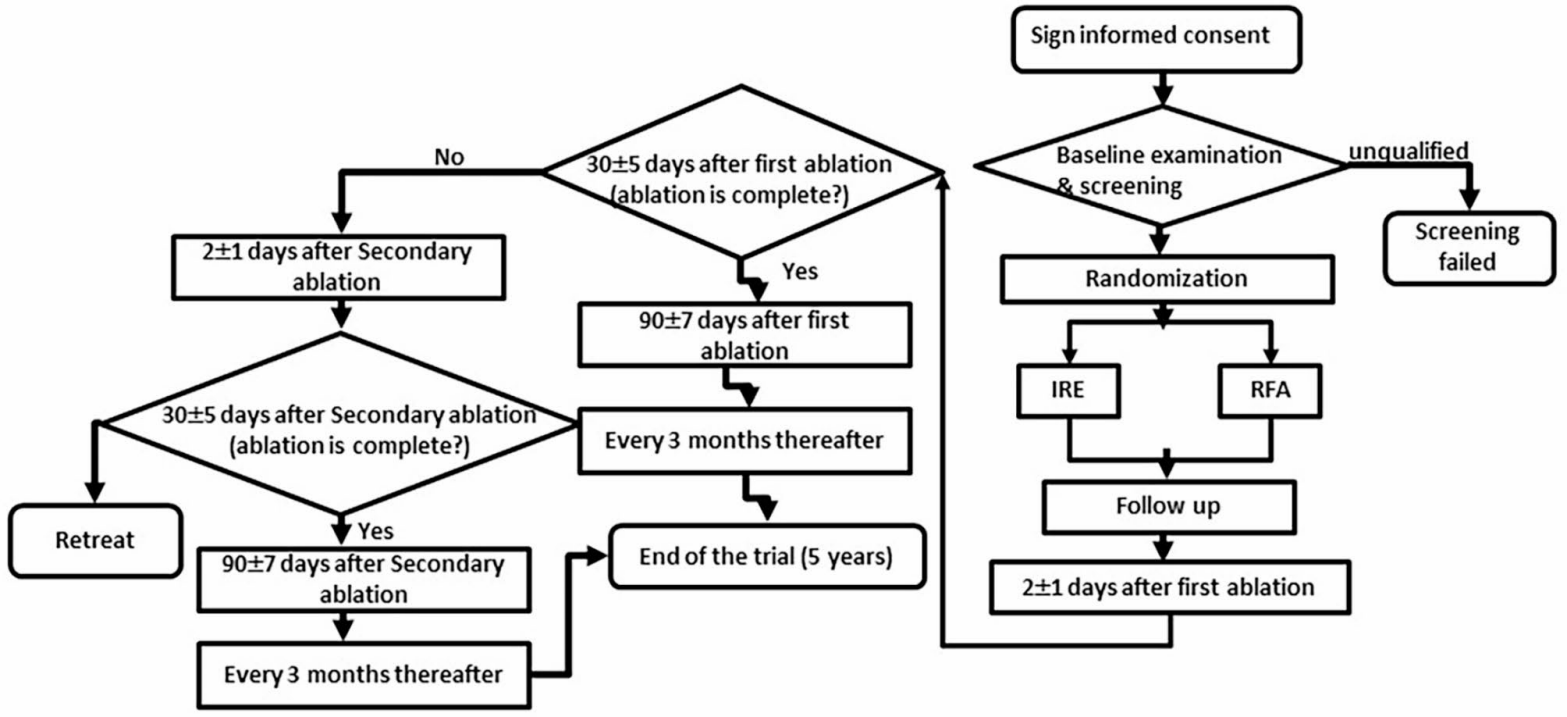
Flow chart of the study

This multicenter, randomized, parallel-controlled clinical trial has been registered on ClinicalTrials. gov (NCT05451160), and has been approved by each ethics committee in accordance with the approval procedures of each trial site (the name of relevant ethics committees which provided approval this study and the reference number and year can been seen in the Supplementary Material). All patients entering the trial screening were required to provide written informed consent. The researchers will strictly follow the protocol and good clinical practice requirements to fully protect the legitimate rights, interests, and safety of the participants. This study protocol follows the standard protocol for clinical trials in accordance with the SPIRIT 2013 statement [12] and follows the CONSORT statement for clinical trial transparency [22, 23]. The study protocol conforms to the ethical guidelines of the 1975 Declaration of Helsinki (6th revision, 2008) as reflected in a priori approval by the institution’s human research committee. The flow chart of the study is shown in Fig. 2.

### Patient recruitment

The participants will be recruited among patients with hepatocellular carcinoma (HCC) at the First Affiliated Hospital of Zhejiang University School of Medicine, the First Affiliated Hospital of Guangxi Medical University, Zhejiang Cancer Hospital, West China Hospital, Tianjin Third Central Hospital, and Third Affiliated Hospital of Sun Yat-Sen University. Before recruitment, the researchers will introduce the two treatments (Irreversible electroporation (IRE) and radiofrequency ablation (RFA)), including their strengths and limitations, to the participants. Written informed consent will be obtained prior to eligibility screening. Throughout the study, participants could decide to withdraw their consent for any reason. Follow-up results will be disseminated to study participants through written reports and telephone exchanges.

### Patient screening

Eligibility screening will be performed within 14 days before procedure, as shown in Table 1 & Suppl. Table 1.

**Table 1:** Follow-up scheme

Trial phase Trial content			a Screening	Follow-up			
			Visit 1	Visit 2	Visit 3	Visit 4	Visit 5
			Preoperative (-14 ~ 0d)	Ablation day (0d)	2 ± 1d after first ablation	30 ± 5d after first ablation	Every 3 months after first ablation
Informed consent^b^			√				
Demographic data			√				
History of liver tumors and past medical history			√				
Vital signs			√	√	√	√	√
Laboratory examination	Urine pregnancy^c^		√				
	Routine urine test^d^		√				
	Routine blood test^e^		√		√	√	√
	Stool routine examination^f^		√				
	Hepatorenal function^g^		√		√	√	√
	Serum electrolyte^h^		√		√	√	√
	Coagulation function^i^		√		√	√	√
	Myocardial enzyme^j^		√		√	√	√
	Tumor marker^k^		√			√	√
Lung CT scan			√				
ECG			√		√	√	√
CEUS			√		√	√	√
Enhanced MRI/CT examination of upper abdomen^l^			√			√	√
ECOG			√			√	√
Child-Pugh			√			√	√
Random allocation				√			
Ablation				√			
Observation of device defects				√			
Evaluation of ablation efficacy						√	√
Record combined medication/treatment			√	√	√	√	√
Handle and record adverse events				√	√	√	√
Verify deviation from protocol				√	√	√	√
Immune index in peripheral blood		The proportion of MDSC ^o^	√	√	√	√	√
		The proportion of TEMs ^o^	√	√	√	√	√
		The concentration of cytokines in serum ^o^	√	√	√	√	√
		Tregs^p^	√	√	√	√	√
		aTregs^r^	√	√	√	√	√
		rTregs^s^	√	√	√	√	√

^(a)^ If the patient has undergone relevant examinations before providing written informed consent and within 14 days before procedure, these data can be collected as data of this clinical trial, and the patient does not need to undergo relevant examinations such as laboratory examination, electrocardiography, and lung CT. The informed process shall be conducted prior to any screening process associated with the trial. ^(b)^ The informed consent process shall be conducted prior to any screening process related to the trial. ^(c)^ Women of childbearing age (18–50 years old and >50 years old without amenorrhea) ^(d)^ Routine urine tests should include at least protein, red blood cell count, and white blood cell count ^(e)^ Routine blood tests should include at least red blood cell count, white blood cell count, hemoglobin, and platelet count ^(f)^ Routine stool tests should include at least occult blood test ^(g)^ Hepatorenal function test should at least include alanine aminotransferase, aspartate aminotransferase, γ−glutamyl transferase, total protein, albumin, total bilirubin, direct bilirubin, blood urea nitrogen or urea, and creatinine ^(h)^ Serum electrolytes should contain at least the following test items: potassium ion, sodium ion, chloride ion, and calcium ion ^(i)^ Coagulation function tests should at least include the prothrombin time, activated partial thromboplastin time, and international normalized ratio ^(j)^ The myocardial enzyme spectrum should at least include phosphocreatine kinase, phosphocreatine kinase isoenzyme, and lactate dehydrogenase ^(k)^ Tumor markers should at least include alpha fetoprotein and carcinoembryonic antigen ^(l)^ Screening MRI/CT examination can still be used within 30 days ^(m)^ Only patients whose first tumor ablation result was “incomplete ablation” should undergo a second ablation, which should be completed within 2 weeks after Visit 4. If Visit 4 and Supplementary visit 1 are completed within the same period of admission, these two visits can be combined as one visit. The inspection performed at Visit 4 is not required for Supplementary visit 1. If not completed within the same admission period, the routine blood and coagulation function tests required at Supplementary visit 1 are available within 14 days before procedure. ^(o)^ These indexes were tested by flow cytometer ^(p)^ Regulatory T cells ^(q)^ Activated regulatory T cells ^(r)^ Resting regulatory T cells CT, computed tomography; ECG, electrocardiography; CEUS, contrast−enhanced ultrasound; MRI, magnetic resonance imaging; CT, computed tomography; ECOG, Eastern Cooperative Oncology Group performance status

The inclusion criteria are as follows:

1. The patient is 18 to 80 years old, and gender is not limited.
2. Contrast enhanced MRI or CT examination of upper abdomen would be performed. For clinically or pathological diagnosed HCC, the diameter of a single tumor is ≤ 5 cm; for multiple tumors, the number of tumors does not exceed three, and the largest tumor diameter is ≤ 3 cm.
3. The Eastern Cooperative Oncology Group (ECOG) performance status ≤ 2.
4. The liver function classification is Child–Pugh A or B.
5. Life expectancy at least 3 months.
6. The patient is able to understand and comply with the trial protocol and provide written informed consent.

The exclusion criteria are as follows:

1. Other treatment < 6 weeks prior to treatment.
2. Transcatheter arterial chemoembolization (TACE).
3. Severe infectious disease such as bacteremia or toxemia.
4. Uncorrectable coagulation dysfunction (platelet count of < 40 × l09/L).
5. Lesions abutting main biliary.
6. Severe heart, brain, lung, or other disease, or the patient has severe arrhythmia.
7. Severe coagulation abnormalities.
8. Implanted artificial hearts, lungs, internal pulse regulators, or wearable medical electronic devices such as electrocardiographic monitors.
9. Has a history of epilepsy.
10. Acute myocardial infarction within 6 months.
11. Pregnant or lactating or plans to become pregnant within 1 year.
12. Allergic to ultrasound, CT, or MRI contrast agents.
13. Contraindications to general anesthesia.
14. The patient has participated in a clinical trial of any drug or medical device within the first 3 months of the present study.
15. Other factors that make the patient unsuitable for inclusion or that affect the patient’s participation in the study.

### Ablation procedure

**Fig. 3:**
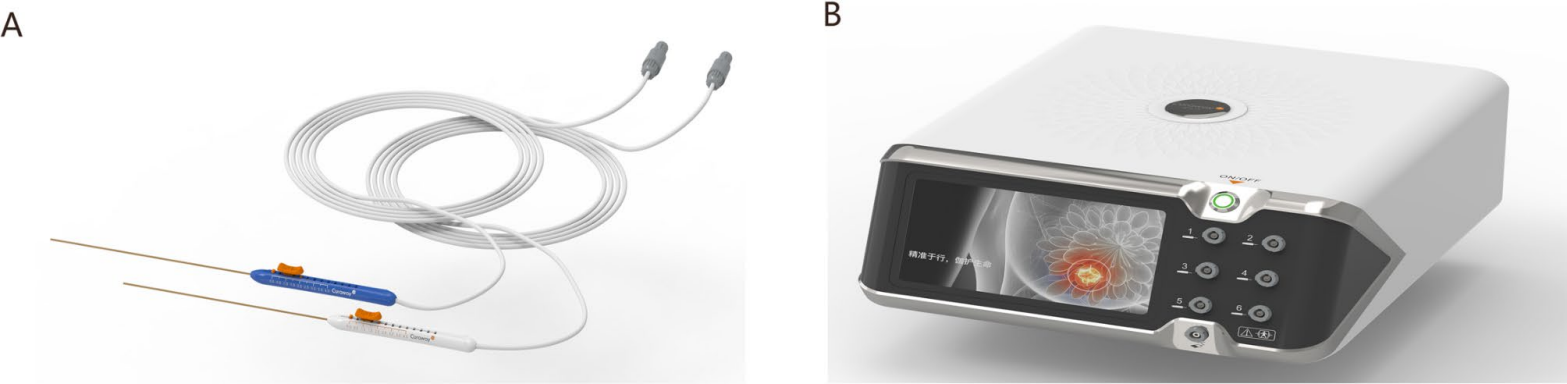
IRE positioning and IRE machine. (**A. B**) electrodes and equipment of IRE. (**C. D. E**) 2–4 electrodes positioning

The patients will be randomly divided into trial arm and control arm. The patients in the trial arm will undergo IRE treatment with the steep pulse therapy system produced by Zhejiang Ganevi Medical Technology Co., Ltd. (Zhejiang, China) (Fig. 3A&B). In the control arm, the RFA system produced by Covidien/Medtronic (Fridley, MN, USA) will be used. Before anesthesia, the patient’s basic information will be checked; the contrast-enhanced ultrasound (CEUS) examination findings will be carefully read; and the radiologist will become familiar with the anatomical location, diameter, and number of lesions and then determine the most appropriate needle placement method. The most appropriate position of procedure will be chosen according to liver segment that lesions located in. Both IRE and RFA were performed percutaneous with ultrasound guidance.

#### IRE procedure

**Fig. 4:**
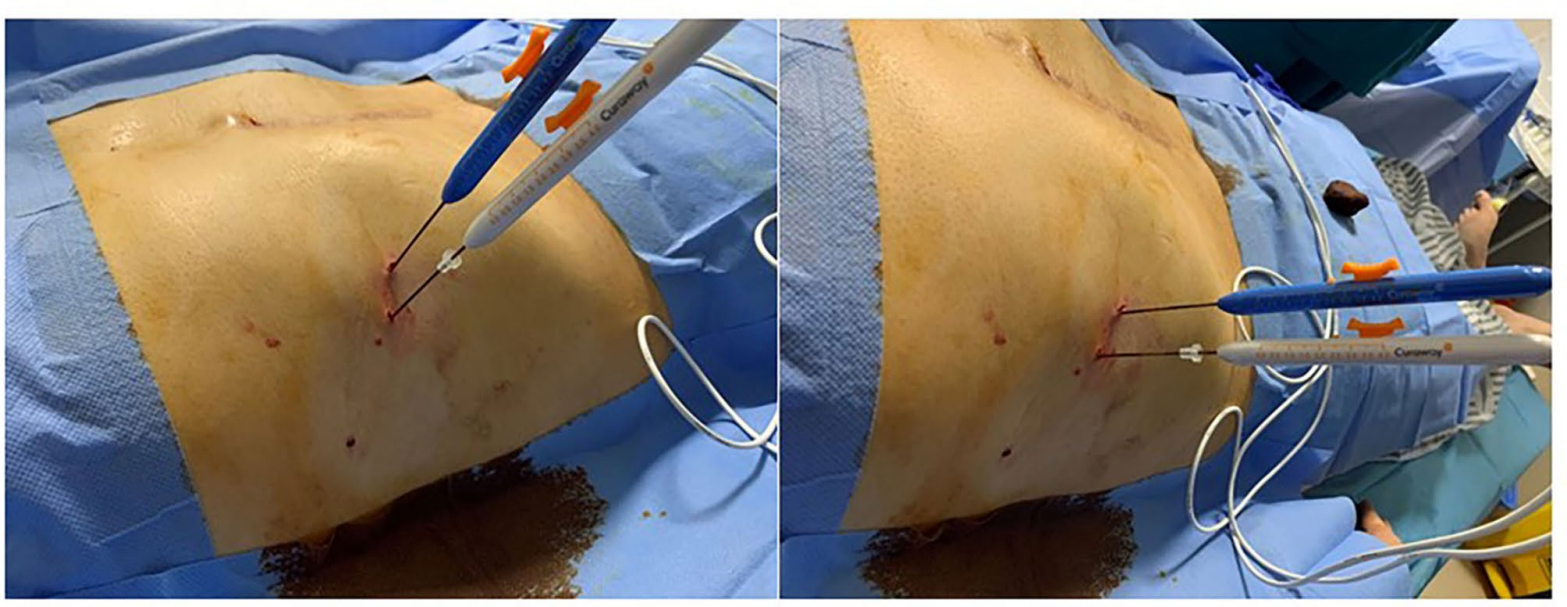
Probe position and patient posture during procedure

**Fig. 5:**
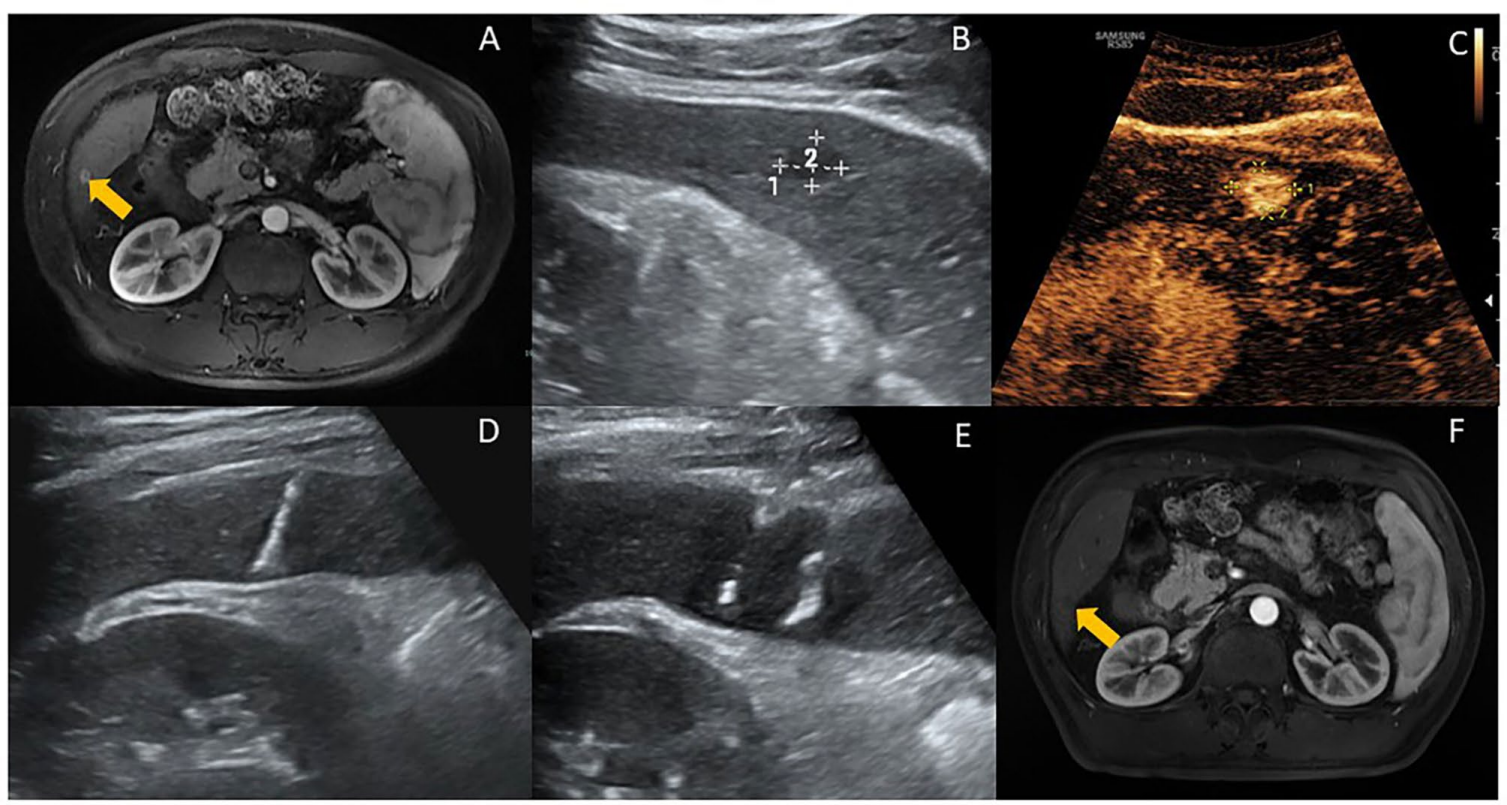
Ultrasound-guided IRE procedure for HCC. (**A**) Pre-ablation contrast-enhanced MRI revealed a hepatocellular carcinoma. (**B&C**) Ultrasound and Contrast-enhanced ultrasound showed a tumor located in S6 (arrow). (**D&E**) Ultrasound-guided IRE with a two-electrode configuration, and the head and tail end of the active tip of the electrodes can have punctuated enhancements (arrow). (**F**) The contrast-enhanced MRI results of one-month after the ablation revealed the tumor shrank obviously and no enhancement after ablation (arrow)

After induction of anesthesia, the shape of the ablation area, the number of electrodes, and the needle puncture routes will be designed according to the size and location of the tumor under ultrasound guidance. A 19G monopole electrode needle (Fig. 3A) will be used for the ablation needle, which will have a length of 15 cm, tip exposure of 1 to 2 cm, and needle distance of 1.0 to 2.5 cm. During the puncture process, the radiologist will ensure that the electrodes are parallel to each other and decide whether to perform layered ablation according to the size of the lesion (Fig. 4). The ablation parameters are as follows: voltage, 2500 to 3000 V; 90 to 120 pulses; and wavelength, 70 to 90 us. These parameters will be adjusted according to the intraoperative current rise; the blood pressure, heart rhythm, and muscle spasms will be monitored; and the pulse will be stopped for corresponding treatment when necessary. The images of the patients’ treatment process are shown in the IRE group (Fig. 5A-F). A video of one patient before, during and after treatment is shown in Video 1.

#### RFA procedure

All RFA procedures were performed by interventional radiologists with at least 2 years of experience. Intravenous sedation and local anesthesia were used for patients who could cooperate with procedure, and general anesthesia was used for patients who were tension or other reasons cannot cooperate. An RF electrode manufactured by Covidien/Medtronic (Fridley, MN, USA) was inserted under ultrasonography guidance.

Following the completion of the RFA and IRE, CEUS will be performed to confirm adequate ablation, defined as an ablation zone that includes the entire target tumor and a safe margin of at least 0.5 cm. If insufficient ablation area is suspected, additional energy deposition cycles for overlapping ablation are performed, preferably after electrode pull-back (partial withdrawal of the needle from 1 to 2 cm along the initial puncture axis) and/or partial or complete electrode re-insertion (in a different axis of the initial puncture).

### Follow-up

The efficacy and safety endpoints will be collected 2 ± 1, 30 ± 5, 60 ± 7 days and every three months after the operation, as shown in Table 1 & Suppl. Table 1. This study had one year of enrollment, and the total trial duration was 3 years. The study will end in May 2025, Since the first subjects were recruited in May 2022. At the end of the trial, 2 independent radiologists will review all images before and after treatment, and reach a consensus. Safety assessment is shown in the Supplementary Materials. Preliminary results can be seen in the Supplementary Materials.

### Study outcomes

#### Primary outcome

The primary outcome is to compare the PFS of IRE to RFA for HCC patients.

#### Key secondary outcomes

The key secondary outcome is to compare the OS of IRE to RFA for HCC patients.

#### Other secondary outcomes

Other secondary outcomes are:

- 30-day complete ablation rate (see Supplementary materials for details evaluation methods).
- 30-day total complete ablation rate (see Supplementary materials for details evaluation methods).
- The safety of IRE and RFA as measured by the rate and severity of AEs.
- The efficacy of IRE compared to RFA on:
- Confirmed Objective Response Rate (ORR);
- Duration of Response (DoR).

### Adverse events

Adverse events refer to unfavorable medical events that occur during clinical trials, regardless of whether they are related to the therapy. The severity of adverse events will be graded according to Common Terminology Criteria for Adverse Events version 5.0. Possible ablation adverse events are shown in the Supplementary Materials.

### Possible cross overs

For patients who were originally assigned to the RFA treatment group but are not suitable for RFA due to factors such as tumor size and location, we will switch to IRE treatment out of ethics and responsibility for the patients. Similarly, for patients who were originally assigned to the IRE treatment group but are not suitable for IRE and are better suited for RFA, for example patients with implanted artificial hearts, lungs, internal pulse regulators, or wearable medical electronic devices such as electrocardiographic monitors, we will switch them to RFA treatment.

### Statistical analysis

#### Calculation of sample size

Assuming a median PFS of 7 months and hazard ratio of 0.347 in the IRE arm based on the results of the previous study [24, 25]. This study had one year of enrollment, and the total trial duration was 3 years. The non-inferiority margin is 20%, which was recognized by clinical experts. α and β are the significance level of statistical tests and test power, respectively, α = 0.05 and β = 0.2 in the present study. Considering a dropout rate of 15% during the study, the number of planned patients in each group was 80. Randomization was performed in a 1:1 ratio, with a total of 190 patients enrolled in the two groups.

#### Statistical methods

The statistical description will be performed using the mean, standard deviation, maximum, minimum, median, confidence interval, and rate (composition ratio). For measurement data conforming to a normal or approximately normal distribution, the t-test for two independent samples, repeated-measures analysis of variance, and analysis of covariance will be used for comparison between the two groups, and the paired t-test will be used for comparison between the pretreatment period and each follow-up time point. When the distribution does not conform to a normal or approximately normal distribution, the Wilcoxon rank sum test will be used for comparison between the two groups, and the paired Wilcoxon rank sum test will be used for comparison between the pretreatment period and each follow-up time point. Categorical data will be compared between the two groups using the chi-square test and Fisher’s exact test. McNamar’s chi-square test will be used for comparison between the pretreatment period and each follow-up time point, and the Cochran–Mantel–Haenszel chi-square test will be used for the central effect. The Wilcoxon rank sum test will be used to compare the rank data between the groups. PFS and OS were summarized with the Kaplan-Meier method. mixed-effects Cox models will be used to analyze factors affecting the efficacy. PFS will be based on BICR assessments of tumor assessment and using mRECIST version 1.1. In our final analysis, we will perform the analysis based on intention to treat, full analysis set, Per-Protocol and Safety Analysis Population analysis set. See Supplementary Materials for detailed analysis sets. Analyses were done using R version 4.2.3, an open-source software developed initially in 1993 by Ross Ihaka and Robert Gentleman at the University of Auckland, New Zealand.

### Publication and presentation plans

After collecting a certain amount of data, we will analyze and organize the final results for publication in relevant journals. We will also submit our research findings to relevant academic conferences, where we will present them orally or as posters to engage in in-depth exchanges and discussions with peers. All datasets collected during the study will be de-identified according to relevant ethical and legal regulations and made accessible through public data repositories to facilitate subsequent research and verification.

## Preliminary results

### Basic characteristics

At the time of this writing, 48 (25.3%) patients had been recruited from 5 centers, of which, 33 patients with 38 tumors had completed the 1-month follow-up and 21 patients have complete the 3-month follow up, with 2.3 months median follow up period. Their basic information is shown in Suppl. Table 2. The patients’ median age was 59.1 years (55.8 years in the Irreversible electroporation (IRE) group and 63 years in the RFA group), and their ages ranged from 29 to 79 years. Seventeen patients have cirrhosis. Only one patient has Child–Pugh grade B disease. The mean longest diameter of the 38 tumors was 1.9 cm and the mean largest tumor diameter is 3.9 cm (Suppl. Table 3).

### Hepatorenal function

Among the 33 patients who were followed up for 1 month, the creatine kinase-MB (CK-MB), alanine aminotransferase (ALT) and aspartate aminotransferase (AST) concentrations were measured before, 2 ± 1 days after, and 30 ± 5 days after the operation (Suppl. Table 4). Both IRE and RFA can cause a large increase in the ALT and AST concentrations shortly after the procedure (*P* < 0.0001 & *P* < 0.0001 for IRE, and *P* = 0.002 & *P* = 0.0007 for RFA); however, it returned to normal one month after procedure, and the effects of IRE and RFA on these two indexes were not significantly different at one month after procedure compared with those before procedure (*P* = 0.634 & *P* = 0.367 for IRE, and *P* = 0.764& *P* = 0.696 for RFA).

### Adverse events

The main adverse event was local pain, which occurred in both IRE group and RFA group (one in RFA group and one in IRE group, both relieved spontaneously within 24 h after procedure). The major adverse event was pleural effusion in one patient in the control group and relieved after pleural effusion catheterization.

### Complete ablation

All 33 patients achieved complete ablation for 1 month (both the IRE and RFA groups had an 100% 1-month complete ablation rate). The images of the patients’ treatment process showed an obvious shrinkage of the lesions in the IRE group (Fig. 5A-F). A video of one patient before, during and after treatment is shown in Video 1.

## Discussion

This is the first randomized controlled clinical trial to compare the efficacy and safety of Irreversible electroporation (IRE) with radiofrequency ablation (RFA) for hepatocellular carcinoma (HCC) patients. Although previous studies compared the effects of IRE and RFA and showed that the safety and efficacy of IRE were greater than those of RFA, these studies were retrospective in nature, preventing control of the consistency of baseline data between the two groups [21]. The tumor size was also significantly different between the IRE group and RFA group, which seriously affected the comparability of the results, and the strength of evidence strength provided by the results was therefore low. In contrast, the present study is a randomized controlled trial. The patient inclusion criteria are being strictly implemented, and a randomized method is being used to divide the groups; thus, the baseline data of the trial group and control group will remain consistent, and selection bias will be reduced. We expect that this study will therefore provide strong evidence for the application of IRE in HCC and provide more choices for the treatment of patients with advanced HCC. In this study, the needle will be placed under ultrasound guidance, allowing the process to be observed and adjusted in real time. This method is being used by increasingly more researchers, and its safety and effectiveness have been proven [26, 27].

This study has a larger sample size than that of previous retrospective studies [21, 28]. In addition, this study is a multicenter clinical trial, thus avoiding the selection bias caused by a single-center design and the information bias caused by differences in medical conditions and doctors’ experience levels. Our study design also increases the representation of the enrolled patients, making the results more reliable. We have begun to recruit participants. At the time of this writing, 48 (25.3%) patients had been recruited from 5 centers. The first patient was enrolled on 23 May 2022 and randomly assigned to the IRE group. Thus far, 28 patients have been treated by IRE and 20 patients have been treated by RFA. Among them, 33 patients completed 1 month of follow-up, and all tumors were completely ablated (complete ablation rate of 100%). And no end point was observed for PFS or OS in both groups, which may be due to the current shorter follow-up period. Further follow-up is needed to obtain more reliable results.

The ALT and AST concentrations increased shortly after procedure in both the IRE and RFA groups, especially IRE groups, but they then decreased 1 month after procedure, and the effect of IRE on these two indices was not significantly different from that of RFA at one month after procedure compared with those before procedure. This may be due to the fact that RFA is thermal ablation, and the corresponding cells are coagulative necrosis, resulting in the release of enzymes cannot into the blood vessels, so the increase of ALT and AST cannot be detected, while IRE is nonthermal ablation, which uses electric pulses to create nanopores in the cell membrane of the tumor cell, eventually causing the cell to undergo apoptosis mainly. Therefore, the enzymes can be released to blood vessels and can be detected thereby. Further follow-up is needed to obtain more reliable results. The complete ablation rate of the two groups was 100%, and there was no statistically significant difference. The lesions of the patients in the IRE group showed obvious shrinkage 1 month after procedure. This suggests that IRE is effective in tumor reduction, but further follow-up is needed to obtain more reliable results.

This study also has its limitations: Firstly, due to the fundamentally different principles and operational methods of IRE and RFA treatments, it is not feasible to blind patients or surgeons during the clinical procedure, which may introduce bias. To minimize this bias, we have adopted blinded data analysis. Additionally, as we are currently in the clinical design stage and have a smaller sample size, we are unable to provide more detailed safety, superiority results, or incorporate more statistical models such as sensitivity or subgroup analyses. After collecting a certain amount of data in the future, we will provide more detailed safety, efficacy results, and additional statistical models.

## Conclusion

This randomized controlled multicenter clinical trial is being performed to comprehensively compare the safety and efficacy of IRE versus RFA in patients with HCC, especially concerning the improvement in the progress free survival time, the overall survival time, the complete ablation rate, the rate of adverse events, and the incidence of adverse events. The results of this study will help to illustrate the clinical value of IRE and provide a beneficial alternative treatment for patients with HCC.

## Electronic supplementary material

Below is the link to the electronic supplementary material.

Supplementary Material 1

Supplementary Material 2

## Data Availability

No datasets were generated or analysed during the current study.
