# Supplementary material for: A multicenter, randomized, parallel-controlled clinical trial protocol to evaluate the safety and efficacy of irreversible electroporation compared with radiofrequency ablation for the treatment of small hepatocellular carcinoma
Source: World J Surg Oncol. 2024 Dec 20;22:332. doi: 10.1186/s12957-024-03614-z (PMC11662571; doi:10.1186/s12957-024-03614-z)
Supplement: Supplementary file 1 — Supplementary Material 1 [file 12957_2024_3614_MOESM1_ESM.docx]

Supplementary Material

**Relevant ethics committees which provided approval this study and the reference number and year.**

**Style: Name of relevant ethics committees/reference year/reference number**

Ethics Committee of the First Affiliated Hospital of Zhejiang University School of Medicine/2022/PRO20220036.

Ethics Committee of the First Affiliated Hospital of Guangxi Medical University/2022/2022-Q004-01.

Ethics Committee of Zhejiang Cancer Hospital/2022/IRB-2022-1026.

Ethics Committee on Clinical Trial.West China Hospital of Sichuan University/2022/HX-IRB-AF-18-V4.0.

Ethics Committee of Tianjin Third Central Hospital/2022/IRB2022-022-01.

Ethics Committee of Third Affiliated Hospital of Sun Yat-Sen University/2022/EL AF/SC-12/03.0.

**Safety assessment**

(1) Vital signs

Blood pressure, heart rate, temperature, and respiration will be measured to assess changes during the trial.

(2) Laboratory tests

Routine urine, blood, and stool indices; liver and kidney function; serum electrolytes; coagulation function; a myocardial enzyme profile; and tumor markers will be examined to evaluate changes during the trial. Suppl. Table2 shows the minimum items that will be included in each test.

(3) Scale assessment

The ECOG score and Child–Pugh score of liver function will be calculated to evaluate the changes during the trial.

(4) Adverse events

Adverse events and serious adverse events will be observed and recorded, and the incidence will be calculated.

Serious adverse events refer to those that occur during the course of a clinical trial and lead to death or serious deterioration of health conditions (including fatal diseases or injuries as well as permanent defects in body structure or function), the need for hospitalization or extension of the hospital stay, the need for medical or surgical intervention to avoid permanent defects in body structure or function, and the possibility of causing fetal distress, fetal death, or congenital abnormalities.

**Complete ablation rate**

Dynamic enhanced MRI/CT of the upper abdomen will be performed to evaluate the efficacy of tumor ablation. MRI will be the first-choice technique, and CT can be used in patients with contraindications to MRI. These imaging examinations should be performed consistently on the same patient throughout the clinical trial. The evaluation criteria are as follows.

- Single lesion
- Complete ablation: Dynamic enhanced CT or MRI shows that the tumor area has low density, and no enhancement is observed in the arterial phase.
- Incomplete ablation: Dynamic enhanced CT or MRI shows local arterial enhancement within the tumor lesion, suggesting residual tumor tissue.
- Multiple lesions
- The ablation effect can only be judged as complete ablation if all the lesions undergoing ablation have reached the standard of complete ablation. Two radiographic diagnostic personnel in each center will be required to perform independent evaluations, which will be separately recorded on different evaluation forms. When the evaluation results of the two personnel are inconsistent, a third person with radiographic diagnostic qualification in the test center will conduct a re-evaluation, and this evaluation result will be the final result. The following formula will be used: complete ablation rate = (total number of patients with complete ablation of target lesions / total number of patients in this group) ×100%.
- Total complete ablation rate (time frame: 90 days): The evaluation method for the total complete ablation rate is the same as that of the primary outcome, and the evaluation will be conducted for all patients who have undergone primary and secondary ablation (secondary ablation will be performed if the primary ablation has been judged incomplete). The following formula will be used: total complete ablation rate = ((number of patients undergoing first complete ablation of target lesions + number of patients undergoing second complete ablation) / total number of patients in this group) ×100%

**Total complete ablation rate**

The evaluation method for the total complete ablation rate is the same as that of the primary outcome, and the evaluation will be conducted for all patients who have undergone primary and secondary ablation (secondary ablation will be performed if the primary ablation has been judged incomplete). The following formula will be used: total complete ablation rate = ((number of patients undergoing first complete ablation of target lesions + number of patients undergoing second complete ablation) / total number of patients in this group) ×100%.

**Possible surgical adverse events in this study and their treatment**

Adverse events of IRE ablation include: bleeding; thrombosis; infection/inflammation; hematoma; pleural effusion; ascites; bile duct injury; pain; arrhythmia; liver abscess; pneumothorax; hemothorax; hypertension; intestinal obstruction; liver failure etc.; the clinician will deal with the subject according to the actual situation of the subject;

The incidence of radiofrequency ablation adverse events is 0-12%, and the incidence of minor adverse events is about 4.7%, mainly including fever, pain, superficial second-degree burns, a small amount of pleural effusion, and a small amount of pneumothorax; the incidence of serious adverse events is about 2.2%., including infection, gastrointestinal bleeding, intra-abdominal bleeding, tumor seeding, liver failure, intestinal perforation, etc.^1^. The clinical symptoms, causes and/or treatment measures of some surgical adverse events are as follows:

1) Pain: Intraoperative pain is mostly mild to moderate, lasting several days to 2 weeks, and can be relieved by analgesic treatment; postoperative pain is mostly mild, and moderate or more pain is rarely seen, and moderate and severe pain is excluded. Sufficient analgesia should be given after acute abdomen, bleeding, etc.

2) Post ablation syndrome: refers to low fever, fatigue, general discomfort, nausea, vomiting and other manifestations that occur transiently after ablation, which are mostly self limited. Its severity and duration are positively related to the size of ablation volume, but there are also individual differences; It generally lasts for 2-7 days, and the ablation volume is large for 2-3 weeks; Symptomatic treatment is given as necessary.

3) Biliary-cardiac reflex: (1) Cause: surgery or heat stimulates the gallbladder system and excites the vagus nerve, resulting in a slow heart rate and a drop in blood pressure. In severe cases, it can lead to myocardial ischemia, arrhythmia, or even cardiac arrest. (2) Treatment: stop treatment immediately and strengthen sedation and analgesia, and give corresponding emergency treatment if necessary. (3) Prevention: Before surgery, drugs can be used to reduce vagus nerve excitability for patients near the biliary system of the tumor; adequate sedation and analgesia during surgery; ablation conditions should start from low temperature/low power and gradually increase to predetermined parameters.

4) Pericardial tamponade: (1) Cause: Radio frequency electrode needle, biopsy needle and other puncture damage to the pericardium. (2) Treatment: a small amount (<100 m1) of pericardial effusion should be closely observed, if there is an increase in the trend, pericardial puncture and drainage should be performed urgently; above the moderate amount (> 100 ml) of pericardial effusion should be urgently pericardial puncture drainage. (3) Prevention: The tumor is adjacent to the heart, and the needle must be punctured step by step to prevent accidental puncture.

5) Bile tumor: (1) Cause: When the ablation volume is large, a bile tumor can be formed, and secondary bacterial infection is liver abscess. (2) Treatment: asymptomatic patients do not need treatment, and the bile tumor continues to enlarge or form a liver abscess that requires puncture aspiration/catheter drainage; Liver abscesses should be drained with antibiotics (sensitive drugs are selected based on the results of pus culture). (3) Prevention: strict aseptic operation, the presence of infection risk factors (diabetes, biliary tract, pancreatic surgery history, especially biliary anastomosis, duodenal papillotomy, bile duct stent implantation, etc.) and ablation volume is larger according to laboratory results Reasonable use of antibiotics.

6) Liver failure: (1) Causes: large volume in a single ablation, infection, massive bleeding, secondary or higher portal vein and/or bile duct branch injury, etc. (2) Treatment: active liver protection, nutritional support, timely treatment of adverse events (anti-infection, abscess drainage, hemostasis, expansion, bile duct drainage, etc.). (3) Prevention: make individualized ablation plan before operation, control the scope of single ablation, avoid injury of large intrahepatic bile ducts and blood vessels during operation, closely monitor the condition after operation, early detection and timely treatment of adverse events.

7) Intrahepatic hematoma, subcapsular and / or intraperitoneal hemorrhage: (1) causes: tear of liver capsule / liver parenchyma, tumor rupture, vascular injury, inadequate needle ablation, etc. (2) Treatment: conservative treatment for a small amount of bleeding; Active arterial bleeding and massive bleeding should be stopped by pulse embolization or ablation in time; Hemorrhagic shock should be treated actively with anti shock therapy, and the pulse should be embolized. If necessary, surgical exploration should be performed. (3) Prevention: avoid large blood vessel puncture tumor, reduce the number of puncture as much as possible, adjust the position of the radiofrequency electrode needle in the liver or leave the liver capsule for re puncture, and fully ablate the needle path after the operation.

8) Pleural effusion: (1) cause: the tumor is adjacent to the diaphragm, the heat energy during the operation and the necrotic tissue after the operation stimulate the pleura. (2) Treatment: conservative treatment of a small amount of pleural effusion, medium to large amount of pleural effusion puncture aspiration or catheter drainage. (3) Prevention: when ablating tumors adjacent to the diaphragm, avoid radiofrequency electrode needle puncture to the diaphragm. It can be combined with chemical ablation or water / gas separation measures to protect the diaphragm. It can also be ablated under laparoscope.

9) Bile duct and / or gallbladder injury: (1) cause: radiofrequency ablation heat damages bile duct and / or gallbladder. (2) Treatment: mild bile duct dilatation does not require treatment; moderate to severe obstructive jaundice should be treated with catheter drainage or cholangioplasty; gallbladder should be removed when the gallbladder is perforated. (3) Prevention: The larger intrahepatic bile duct adjacent to the lesion can be combined with chemical ablation, or bile duct cannulation can be performed before operation, and normal saline can be continuously pumped through the inserted pipeline to protect it; the lesion adjacent to the gallbladder can be combined with chemical ablation or the gallbladder is protected by water/gas separation and can also be ablated laparoscopically.

10) Hepatic artery-portal vein/hepatic vein fistula: (1) Cause: Puncture injury of hepatic artery and portal/hepatic vein branch. (2) Treatment: Those with small shunt flow do not need treatment, and those with large shunt flow need to use coils to seal the fistula. (3) Prevention: According to the preoperative and intraoperative images, avoid radiofrequency electrode needle puncture to damage the larger arteries and branches of the portal/hepatic vein in the liver.

11) Gastrointestinal tract injury: (I) cause: the tumor is adjacent to the gastrointestinal tract, and radiofrequency ablation heat damages the gastrointestinal tract. (2) Treatment: Patients with gastrointestinal perforation should be treated by gastrointestinal decompression, fasting and timely surgery. (3) Prevention: Patients with suspected gastrointestinal tract invasion should be examined by gastroscope / enteroscope before operation, and patients with gastrointestinal tract invasion should not be treated by radiofrequency ablation; If the tumor is adjacent to the gastrointestinal tract, the intestinal tract should be fully cleaned before operation and fasting and water for more than 24 hours; Precise positioning and reasonable setting of ablation parameters during the operation can be combined with chemical ablation or water / gas separation measures to protect the gastrointestinal tract, and can also be performed under laparoscopy.

12) Diaphragmatic injury: (1) cause: thermal injury of the diaphragm during ablation of tumors adjacent to the diaphragm. (2) Treatment: in case of pneumothorax or pleural effusion, the treatment method is the same as that of "pneumothorax" and "pleural effusion". (3) Prevention: the same as the prevention of pleural effusion.

13) Tumor implantation: (I) Cause: repeated puncture of the tumor and insufficient needle tract ablation. (2) Treatment: ablation treatment. (3) Prevention: Minimize the number of tumor punctures; if the radiofrequency electrode needle has entered the tumor but needs to be adjusted, it must be ablated in situ and then withdrawn for adjustment.

14) Skin burns: (1) Skin burns at the place where the negative plate is pasted: ①Cause: The use of a single negative plate, the poor adhesion of the negative plate, the falling off of one negative plate, etc., cause the local current load at the place where the negative plate is pasted to be too large. ②Treatment: For mild skin scalds, keep the area clean and dry to prevent infection, or apply scalding cream locally; for moderate and severe skin scalds, treat them as burns, with debridement and skin grafting if necessary. ③Prevention: The negative plate is in full contact with the skin, and the paste is dense and symmetrical; when the local hair is thick, it needs to be shaved; the local ice pack of the negative plate is cooled; if one side of the negative plate is overheated, the cause should be found immediately.(2) Skin scald at the skin puncture point: ① Cause: excessive ablation of the needle tract. ②Treatment: Keep the area clean and dry to prevent infection, and apply scald cream locally if necessary. ③ Prevention: Avoid excessive ablation of the needle tract.

15) Rare adverse events include intercostal artery and intercostal nerve injury, bile duct and bronchial fistula, etc.

**Analysis sets**

**Full analysis set (FAS)**

The FAS will include all patients randomized into the study. The FAS will be the primary analysis set for all efficacy analyses. Following the intent-to-treat principle, the patients will be analyzed according to the treatments and strata to which they were assigned at randomization.

**Safety analysis set**

The safety analysis set will include all randomized patients who underwent ablation surgery, either IRE or RFA. The patients will be summarized according to the treatment actually received. The treatment received is the randomized treatment unless the alternative treatment is received throughout the study.

**Per-protocol analysis set (PPS)**

The PPS will include all patients in the FAS who sufficiently complied with the protocol in terms of the exposure to study treatment, availability of tumor assessments, and absence of major protocol deviations likely to impact the efficacy outcome.

Efficacy analyses will be performed on the FAS and PPS. All baseline demographic data analyses will be performed on the FAS.

**Multiple comparisons/multiplicity**

The potential source of multiplicity in this trial will be the multiple group comparisons. To address this multiplicity issue, the Bonferroni method will be used to adjust the value of α to control the type I error rate.

**Suppl. Table1** Follow-up scheme of secondary ablation

| Trial phase  Trial  content | | |  | | | | |
| --- | --- | --- | --- | --- | --- | --- | --- |
|  |  |  | Supplementary visit 1^m^ | Supplementary visit 2 | Supplementary visit 3 | Supplementary visit 4 | Supplementary visit 5 |
|  |  |  | Secondary ablation day (0d) | 2±1d after Secondary ablation | 30±5d after Secondary ablation | 90±7d after Secondary ablation | Every 3 months after Secondary ablation |
| Informed consent^b^ | | |  |  |  |  |  |
| Demographic data | | |  |  |  |  |  |
| History of liver tumors and past medical history | | |  |  |  |  |  |
| Vital signs | | | √ | √ | √ | √ | √ |
| Laboratory examination | Urine pregnancy^c^ | |  |  |  |  |  |
|  | Routine urine test^d^ | |  |  |  |  |  |
|  | Routine blood test^e^ | | √ | √ | √ | √ | √ |
|  | Stool routine examination^f^ | |  |  |  |  |  |
|  | Hepatorenal function^g^ | | √ | √ | √ | √ | √ |
|  | Serum electrolyte^h^ | | √ | √ | √ | √ | √ |
|  | Coagulation function^i^ | | √ | √ | √ | √ | √ |
|  | Myocardial enzyme^j^ | | √ | √ | √ | √ | √ |
|  | Tumor marker^k^ | |  |  | √ | √ | √ |
| Lung CT scan | | |  |  |  |  |  |
| ECG | | | √ | √ | √ | √ | √ |
| CEUS | | | √ | √ | √ | √ | √ |
| Enhanced MRI/CT examination of upper abdomen^l^ | | |  |  | √ | √ | √ |
| ECOG | | |  |  | √ | √ | √ |
| Child-Pugh | | |  |  | √ | √ | √ |
| Random allocation | | |  |  |  |  |  |
| Ablation ~~surgery~~ | | | √ |  |  |  |  |
| Observation of device defects | | | √ |  |  |  |  |
| Evaluation of ablation efficacy | | |  |  | √ | √ | √ |
| Record combined medication/treatment | | | √ | √ | √ | √ | √ |
| Handle and record adverse events | | | √ | √ | √ | √ | √ |
| Verify deviation from protocol | | | √ | √ | √ | √ | √ |
| Immune index in peripheral blood | | The proportion of MDSC ^o^ | √ | √ | √ | √ | √ |
|  |  | The proportion of TEMs ^o^ | √ | √ | √ | √ | √ |
|  |  | The concentration of cytokines in serum ^o^ | √ | √ | √ | √ | √ |
|  |  | Tregs^p^ | √ | √ | √ | √ | √ |
|  |  | aTregs^r^ | √ | √ | √ | √ | √ |
|  |  | rTregs^s^ | √ | √ | √ | √ | √ |

**Suppl.Table2. Baseline characteristics of 33 patients**

| Characteristic | IRE group | RFA group |
| --- | --- | --- |
| Age (yr) (median, range) | 56(29,79) | 59(47,74) |
| Male gender | 14 (77.7%) | 14 (93.3%) |
| Child-Pugh-Turcotte classification (A) | 17(94.4%) | 15(100%) |
| Tumor number | 21 | 17 |
| Tumor size (Longest Diameter) |  |  |
| ≤2 | 12 | 10 |
| ＞2 | 9 | 7 |
| Tumor size (mean, range) | 2.0 (0.8,3.7) | 1.9(0.7,3.9) |
| Cirrhosis No | 9 (50%) | 7 (46.7%) |
| History of hepatitis |  |  |
| No | 5 (27.8%) | 6 (40%) |
| Hepatitis b | 11 (61.1%) | 9 (60%) |
| Hepatitis c | 1(5.5%) | 0 (0.0%) |
| Hepatitis e | 1(5.5%) | 0 (0.0%) |
| Location |  |  |
| S2 | 4(19.0%) | 0 (0.0%) |
| S4 | 3(14.3%) | 3 (17.6%) |
| S5 | 4(19.0%) | 4 (23.5%) |
| S6 | 2(9.5%) | 3 (17.6%) |
| S7 | 3(14.3%) | 4 (23.5%) |
| S8 | 5(23.8%) | 2 (11.8%) |

**Suppl.Table 3 Minimum laboratory examination items**

| **Check major items** | **The minimum inspection items to be included** |
| --- | --- |
| Routine urine test | Urine protein (PRO), urine red blood cell count (RBC), urine white blood cell count (WBC) |
| Routine blood test | Red blood cells (RBC), white blood cells (WBC), hemoglobin (Hb), platelets (PLT) |
| Stool routine examination | Fecal occult blood test (OBT) |
| Hepatorenal function | Alanine aminotransferase (ALT), aspartate aminotransferase (AST), γ-glutamyl transferase (γ-GT), total protein (TP), albumin (Alb), total bilirubin (TBIL), direct bilirubin (DBIL), urea nitrogen (BUN) or urea (UREA), creatinine (Cr) |
| Serum electrolyte | Potassium (K+), Sodium (Na+), Chloride (Cl-), Calcium (Ca2+) |
| Coagulation function | Prothrombin Time (PT), Activated Partial Thromboplastin Time (APTT), International Normalized Ratio (INR) |
| Myocardial enzyme | Phosphocreatine kinase (CK), Phosphocreatine kinase isoenzyme (CK-MB), Lactate dehydrogenase (LDH) |
| Tumor marker | Alpha-fetoprotein (AFP), carcinoembryonic antigen (CEA) |

**Suppl.Table 4 AST, ALT and CK-MB results at different times**

| Group | CK-MB (U/L) | | | ALT(U/L) | | | AST(U/L) | | |
| --- | --- | --- | --- | --- | --- | --- | --- | --- | --- |
|  | Before | 2±1d | 30±5d | Before | 2±1d | 30±5d | Before | 2±1d | 30±5d |
| IRE | 13 | 26 | 9 | 13 | 115 | 14 | 16 | 139 | 18 |
| IRE | 23 | 25 | 24 | 18 | 129 | 19 | 32 | 85 | 31 |
| IRE | 10 | 14 | 15 | 33 | 56 | 31 | 25 | 37 | 22 |
| IRE | - | 20 | 19 | 47 | 514 | 111 | 21 | 773 | 63 |
| IRE | 42 | 44 | 40 | 15 | 105 | 14 | 23 | 133 | 19 |
| IRE | 10 | 17 | 8 | 7 | 248 | 8 | 17 | 310 | 12 |
| IRE | 65 | 94 | - | 22 | 130 | 43 | 31 | 276 | 54 |
| IRE | 14 | 46 | 11 | 44 | 274 | 34 | 32 | 311 | 28 |
| IRE | 6 | 28 | 13 | 16 | 50 | 14 | 17 | 75 | 17 |
| IRE | 18 | 19 | 22 | 33 | 220 | 27 | 32 | 226 | 27 |
| IRE | 8 | 16 | 9 | 24 | 127 | 24 | 30 | 239 | 28 |
| IRE | 40 | 38 | 40 | 24 | 482 | 20 | 24 | 423 | 24 |
| IRE | 10 | 35 | 9 | 18 | 685 | 26 | 20 | 686 | 25 |
| IRE | 17 | 23 | 17 | 79 | 523 | 49 | 53 | 535 | 50 |
| IRE | 17 | 18 | 16 | 37 | 502 | 35 | 27 | 493 | 29 |
| IRE | 14 | 20 | 9 | 17 | 206 | 24 | 19 | 261 | 20 |
| IRE | 16 | 28 | 35 | 98 | 253 | 132 | 34 | 272 | 52 |
| IRE | 23 | 14 | 15 | 19 | 248 | 22 | 20 | 263 | 21 |
|  |  |  |  |  |  |  |  |  |  |
| RFA | 28 | 28 | 32 | 17 | 72 | 25 | 23 | 86 | 29 |
| RFA | 56 | 57 | 56 | 47 | 61 | 27 | 55 | 79 | 37 |
| RFA | 9 | - | 33 | 33 | 88 | 35 | 31 | 111 | 31 |
| RFA | 21 | 22 | 21 | 27 | 43 | 24 | 33 | 73 | 31 |
| RFA | 23 | 16 | 16 | 24 | 58 | 33 | 23 | 73 | 29 |
| RFA | 41 | 17 | 10 | 74 | 229 | 38 | 61 | 23 | 30 |
| RFA | 13 | 15 | 16 | 21 | 69 | 27 | 22 | 89 | 23 |
| RFA | 8 | 10 | 13 | 26 | 195 | 20 | 36 | 272 | 22 |
| RFA | 14 | 5 | 12 | 30 | 41 | 39 | 37 | 75 | 47 |
| RFA | 24 | 33 | - | 37 | 42 | 46 | 41 | 68 | 51 |
| RFA | 14 | 13 | 14 | 20 | 75 | 17 | 22 | 117 | 22 |
| RFA | 16 | 32 | 14 | 55 | 159 | 47 | 31 | 260 | 27 |
| RFA | 19 | 29 | 29 | 16 | 65 | 19 | 24 | 142 | 25 |
| RFA | 15 | 12 | 61 | 23 | 105 | 28 | 23 | 126 | 31 |
| RFA | 12 | 14 | 14 | 6 | 24 | 7 | 11 | 53 | 13 |

**References**

(1) Izzo F, Granata V, Grassi R, et al. Radiofrequency Ablation and Microwave Ablation in Liver Tumors: An Update. Oncologist 2019;24(10):e990-e1005.
